# Octocog alfa在中国血友病A患者中的临床疗效和安全性：AHEAD研究1年随访结果

**DOI:** 10.3760/cma.j.cn121090-20241022-00410

**Published:** 2025-08

**Authors:** 润晖 吴, 振宇 李, 竞 孙, 新 杜, 心声 张, 缨 王, 群 胡, 荣富 周, Joan Gu, Randy Guerra, 仁池 杨

**Affiliations:** 1 首都医科大学附属北京儿童医院，北京 100045 Beijing Children's Hospital, Capital Medical University, Beijing 100045, China; 2 徐州医科大学附属医院，徐州 221132 The Affiliated Hospital of Xuzhou Medical University, Xuzhou 221132, China; 3 南方医科大学南方医院，广州 515399 Nanfang Hospital, Nanfang Medical University, Guangzhou 515399, China; 4 深圳市第二人民医院/深圳大学第一附属医院，深圳 518035 Shenzhen Second People's Hospital, The First Affiliated Hospital of Shenzhen University, Shenzhen 518035, China; 5 山东省血液中心，济南 250013 Shandong Blood Center, Jinan 250013, China; 6 深圳市儿童医院，深圳 518000 Shenzhen Children's Hospital, Shenzhen 518000, China; 7 华中科技大学同济医学院同济医院，武汉 430030 Tongji Hospital, Tongji Medical College of Huazhong University of Science and Technology, Wuhan 430030, China; 8 南京鼓楼医院/南京大学医学院附属鼓楼医院，南京 210008 Nanjing Drum Tower Hospital, The Affiliated Hospital of Nanjing University Medical School, Nanjing 210008, China; 9 武田美洲开发中心，美国马萨诸塞州剑桥市 Takeda Development Center Americas, Inc., Cambridge, MA, USA; 10 中国医学科学院血液病医院（中国医学科学院血液学研究所），天津 300020 Institute of Hematology and Blood Diseases Hospital, Chinese Academy of Medical Sciences and Peking Union Medical College, Tianjin 300020, China

**Keywords:** 重组人凝血因子Ⅷ, 血友病A, 临床疗效, 1年随访, Recombinant human coagulation factor Ⅷ, Hemophilia A, Clinical efficacy, 1-year follow-up

## Abstract

**目的:**

评估AHEAD研究（NCT02078427）中接受重组人凝血因子Ⅷ（Octocog alfa）治疗的中国血友病A（HA）患者的长期疗效与安全性。

**方法:**

AHEAD研究中国组患者入组截止至2021年1月，数据统计截止至2022年7月15日，主要评估指标包括吉尔伯特（Gilbert）评分、血友病关节健康评分（HJHS）∶总体步态评分、年化出血率（ABR）、年化关节出血率（AJBR）和不良事件（AE）。

**结果:**

本研究纳入168例HA患者，其中113例患者接受预防性治疗，53例接受按需治疗。患者平均年龄为（21.40 ± 13.37）岁，均为男性。与基线相比，预防性治疗组中间型HA患者1年随访期的HJHS：全球步态评分显著降低（*P*＝0.01）；按需治疗组患者HJHS∶总体步态评分显著降低（*P*＝0.008）；预防性治疗组患者平均Gilbert评分的降低幅度高于按需治疗组（28.6％对18.2％）。预防性治疗组HA患者1年随访期ABR评分的平均值显著降低（3.70对7.78，*P*＝0.01），尤其是重型HA患者（2.14对8.98，*P*＝0.006）和儿童HA患者（2.1对4.73，*P*＝0.03）。接受预防性治疗的中剂量组HA患者1年随访期ABR评分显著降低（*P*＝0.015）。1年随访期内25例患者（14.9％）报告了39次不良事件，仅1例患者报道FⅧ抑制物与治疗相关。

**结论:**

接受Octocog alfa预防性和按需治疗HA患者的关节活动能力均有改善。接受预防性治疗的患者出血情况的改善具有统计学显著性，尤其是重型HA患者和儿童HA患者。

## Abstract (en)

**Objective:**

To evaluate the long-term efficacy and safety of recombinant coagulation factor Ⅷ（Octocog alfa）in Chinese patients with hemophilia A（HA）enrolled in the International Antihemophilic Factor Hemophilia A Outcome Database（AHEAD）study（NCT02078427）.

**Methods:**

Enrollment of Chinese patients in the AHEAD study was completed by January 2021, and data were collected up to July 15, 2022. This study primarily assessed patients in terms of the Gilbert score, global gait score within the Hemophilia Joint Health Score（HJHS）, annualized bleeding rate（ABR）, annualized joint bleeding rate, and adverse events.

**Results:**

A total of 168 male patients were included in this study, of which 113 received prophylactic treatment and 53 received on-demand treatment. The average age of the patients was 21.4±13.37 years. Compared with baseline, the global gait score within HJHS significantly decreased during the 1-year follow-up in patients with moderately severe HA in the prophylactic treatment group（*P*＝0.01）and on-demand treatment group（*P*＝0.008）. The mean reduction in Gilbert score was greater in the prophylactic treatment group than in the on-demand treatment group（28.6％ *vs* 8.2％）. The average ABR decreased significantly during the 1-year follow-up（3.70 *vs* 7.78, *P*＝0.01）in the prophylactic treatment group, particularly in patients with severe HA（2.14 *vs* 8.98, *P*＝0.006）and pediatric patients（2.1 *vs* 4.73, *P*＝0.03）. The ABR score also decreased significantly in the moderate-dose prophylactic treatment group（*P*＝0.015）. During the 1-year follow-up, 25 patients（14.9％）reported 39 adverse events, with only one patient developing treatment-related FⅧ inhibitor.

**Conclusion:**

Joint mobility improved in patients receiving either prophylactic or on-demand Octocog alfa. Bleeding episodes significantly reduced in patients receiving prophylactic treatment, particularly in pediatric patients and those with severe HA.

血友病A（HA）是一种X连锁隐性出血性疾病，主要由编码凝血因子Ⅷ（FⅧ）的基因发生突变，导致患者血液中缺乏FⅧ而引起不同程度的出血症状，例如组织、肌肉和关节出血等，通常为男性发病[1]–[2]。流行病学的系统评价显示我国HA的患病率为每10万人2.73～3.09例，年死亡率为0.2％～1％[3]–[4]。中间型［FⅧ促凝活性（FⅧ∶C）1％～5％］和轻型（FⅧ∶C>5％～<40％）HA患者，其出血症状通常与受伤和手术有关，而重型（FⅧ∶C<1％）HA患者可频繁发生关节或肌肉自发性出血或因受伤导致过度出血[5]。出血症状未得到有效控制会导致患者出现更为严重的并发症（如血友病关节病）[6]。目前，使用FⅧ制剂的替代疗法（预防性治疗或按需治疗）是HA患者出血症状的标准疗法[7]。世界血友病联合会（WFH）指南指出预防性治疗是重型和部分中间型血友病患者的标准治疗方法，所有重型HA患者应在关节疾病发生之前（儿童时期<3岁）开始使用凝血因子产品进行预防性治疗。对于所有肌肉骨骼出血的患者，应尽早使用凝血因子替代疗法以实现患者功能恢复[8]。相比血浆来源FⅧ产品，重组FⅧ（rFⅧ）的血浆半衰期更长，止血效果更持久[9]–[10]。国际AHEAD临床研究（NCT02078427）是一项正在进行的大型前瞻性和非干预性多中心真实世界研究，旨在评估重组人凝血因子Ⅷ（Octocog alfa）在HA患者中的长期疗效和安全性。本文汇报AHEAD研究中接受Octocog alfa治疗的中国HA患者的一年随访结果。

## 病例与方法

一、研究设计

Octocog alfa是基于第三代重组基因构建的标准半衰期重组人凝血因子Ⅷ。AHEAD研究开始（首例患者入组）至研究结束（末例患者末次随访），预计研究总持续时间为7年，入组时间截止至2021年1月。本研究汇报了截止至2022年7月15日AHEAD研究中来自中国9个中心接受Octocog alfa治疗的HA患者1年随访结果。

二、纳入和排除标准

1. 纳入标准：①无论先前是否接受过FⅧ产品治疗（预防或按需）的患者（FⅧ∶C≤5％）；②患者需按照经治医师的医嘱使用Octocog alfa治疗；③患者本人或其法定代理人签署知情同意书。

2. 排除标准：①已知对Octocog alfa有超敏反应；②已知对小鼠或仓鼠蛋白有过敏反应；③患者在研究入组前30 d内参与涉及研究用药品（IP）或医疗器械的其他临床研究，或者在本研究过程中计划参与涉及另一种FⅧ浓缩物或医疗器械的其他临床研究；④有人类免疫缺陷病毒（HIV）史、任何血栓栓塞事件或血小板计数≥50 000个/µl的患者。

三、患者招募

本研究纳入168例患者，其中113例接受预防性治疗，53例接受按需治疗，1例患者接受免疫抑制诱导（ITI）治疗，1例患者治疗情况不详。采集患者在入组（基线）和1年随访周期的结局相关指标数据进行分析和统计。试验开始前，66.1％（111例）的患者有超过50个FⅧ产品的暴露天数（ED），47.0％（79例）的患者无抑制物发生史，53.0％（89例）的患者抑制物发生情况不详。患者在参与试验时可进行小手术，需要进行大手术的患者被排除。若患者在随访期间出现出血或进行小手术，则给予额外剂量的Octocog alfa。Octocog alfa允许的最大日总剂量为500 IU/kg[11]。治疗期间有24例患者完成了治疗方案转换，原因包括患者体重增加、病情好转、活动量增加、需要预防性治疗、医生建议和患者经验等。在统计各结局指标时仅评估接受Octocog alfa治疗且未进行治疗方案转换的患者。本研究获得中国医学科学院血液病医院伦理委员会批准（QT2018007-EC-1）并按照《赫尔辛基宣言》及相关伦理要求开展。所有患者入组前均已获得知情同意并签署知情同意书。

四、结局指标

本研究主要疗效终点为通过吉尔伯特（Gilbert）量表[12]评估患者关节活动能力，次要疗效终点包括年化出血率（ABR）、年化关节出血率（AJBR）、血友病关节健康评分（HJHS）：总体步态评分。本研究的安全性结局指标为不良事件（AE）和严重不良事件（SAE）发生率，未单独统计预防和按需治疗组的AE发生率。本研究将患者分为预防和按需治疗组，在每个组中分别统计中间型（FⅧ≥1％～≤5％）和重型（FⅧ<1％）HA患者的疗效指标。

五、数据收集

AHEAD研究中，经治医师将在基线时参考Octocog alfa药品说明书，根据患者年龄和疾病严重程度确定治疗方案及实验室、影像学和临床监测的频率。入组时，每例患者将获得一份患者日记，以协助标准化数据收集。在研究参与期间，患者日记中记录的所有数据，包括AE、出血次数、给药情况等，将会在患者随访时由经治医师录入病例报告表。研究管理人员定期提醒所有患者保持记录的完整性和治疗依从性。患者可自愿撤回继续参与研究和数据收集的知情同意书。已收集的退出研究患者的数据将在分析中使用并纳入临床研究报告。所有患者在Octocog alfa治疗前进行抑制物筛查试验。阴性抑制物标准为通过Nijmegen改良Bethesda检测法测得的任何滴度<0.6 Bethesda单位（BU），或通过非Nijmegen改良Bethesda检测法测得的任何滴度<0.6 BU，低滴度抑制物标准为≥0.6 BU并且<5 BU，高滴度抑制物标准为≥5 BU。

六、统计学处理

本研究采用负二项分布的广义线性混合效应模型（GLMM）进行事后ABR和AJBR分析，并使用正态逼近法对95％置信区间进行估计[13]–[14]，以解释其随访期间的变化。组间两两比较采用Student *t*检验，*P*<0.05为差异有统计学意义。本研究属于非干预性研究，一年随访期时有一定量的数据缺失，分析将使用可用数据进行。只有在基线日期之后的AE被纳入本分析。AE分类基于MedDRA标准[15]，由于单个AE被逐字拆分为几个MedDRA编码，可能导致比实际更高的AE数量。

## 结果

一、患者资料统计

AHEAD研究中国组纳入的168例患者中，94例（55.9％）患者完成了1年随访，34例患者在不满1年研究期内自愿退出/中止研究。168例患者均为男性，其中113例接受预防性治疗，53例接受按需治疗，接受ITI治疗的1例患者和治疗情况不详的1例患者均不纳入分析。患者平均年龄为（21.40±13.37）岁，33.1％（55例）的患者为儿童，13.9％（23例）的患者为青少年，53.0％（88例）的患者为成人。重型患者占比为47.6％（79例），中间型患者占比为50.6％（84例）。12.8％（10例）的患者存在内含子22倒位。在基线时，8.4％（14例）的患者有抑制物发生史，11.9％（10例）的患者开始治疗时抑制物滴度值超出正常范围。预防性治疗组有40.7％的患者目标靶关节数≥1，按需治疗组有83.0％的患者目标靶关节数≥1（表1）。

**表1:** AHEAD研究中国组168例血友病A患者基线临床特征

指标	预防性治疗 （113例）	按需治疗 （53例）
年龄（岁，*x*±*s*）	18.5±13.1	28.0±11.7
年龄分组［例数（％）］		
2岁～<12岁	52（46.0）	3（5.7）
12岁～<18岁	13（11.5）	10（18.9）
≥18岁	48（42.5）	40（75.5）
疾病严重程度［例数（％）］		
重型	60（53.1）	19（35.9）
中间型	51（45.1）	33（62.3）
未分级	2（1.8）	1（1.9）
内含子22倒位阳性［阳性例数/检测例数（％）］	9/55（16.4）	1/23（4.3）
治疗持续时间（d，*x*±*s*）	426.3±195.9	496.9±171.3
暴露天数（d，*x*±*s*）	190.0±193.4	123.8±49.8
有抑制物发生史［例数（％）］	12（10.6）	2（3.8）
开始治疗时FⅧ抑制物情况［阳性例数/检测例数（％）］		
阴性	21/30（70.0）	1/6（16.7）
低滴度	2/30（6.7）	0/6（0）
高滴度	0/30（0）	0/6（0）
未知	7/30（23.3）	5/6（83.3）
基线靶关节数［例数（％）］		
0	67（59.3）	9（17.0）
1	18（15.9）	12（22.6）
2	7（6.2）	12（22.6）
>2	21（18.6）	20（37.7）

**注** FⅧ：凝血因子Ⅷ；重型：FⅧ活性<1％，中间型：FⅧ活性1％～5％

二、患者输注情况

预防性治疗组患者在基线时均获得Octocog alfa治疗方案（每周给药1～7次不等），显示重型HA患者的平均输注剂量高于中间型HA患者，儿童HA患者的平均输注剂量高于青少年和成人HA患者（表2）。在随访1年时，预防性治疗组、按需治疗组分别收集到61、17例患者的Octocog alfa应用数据。1年随访期内，预防性治疗组重型HA患者（47.1 IU/kg）的Octocog alfa平均给药剂量高于中间型HA患者（44.2 IU/kg）；无论是预防性治疗组，还是按需治疗组，儿童HA患者的Octocog alfa平均给药剂量均高于青少年和成人患者（表3）。

**表2:** 基线时预防治疗组不同疾病严重程度和年龄患者的凝血因子Ⅷ（FⅧ）每周输注量（IU/kg，*x*±*s*）

组别	例数	FⅧ每周输注量
重型	55	46.4±21.7
中间型	48	42.8±18.9
2岁～<12岁	50	47.3±22.2
12岁～<18岁	13	42.8±25.6
≥18岁	41	41.7±16.0

**注** 重型：FⅧ活性<1％；中间型：FⅧ活性1％～5％

**表3:** 1年随访期时不同疾病严重程度和年龄组患者的Octocog alfa每周输注量（IU/kg，*x*±*s*）

组别	预防性治疗组（61例）	按需治疗组（17例）
重型	47.1±17.4	4.5±7.1
中间型	44.2±19.9	13.0±23.8
2岁～<12岁	54.8±18.4	27.9±44.4
12岁～<18岁	38.9±8.9	3.2±0.8
≥18岁	43.4±19.9	5.9±6.9

**注** 重型：FⅧ活性<1％；中间型：FⅧ活性1％～5％

三、关节活动能力

如表4所示，与基线相比，预防性治疗组患者1年随访期最大Gilbert评分、平均Gilbert评分和HJHS∶总体步态评分均降低；预防性治疗组中间型HA患者1年随访期的HJHS∶总体步态评分显著降低（*P*＝0.01）。与基线相比，按需治疗组1年随访期最大Gilbert评分、平均Gilbert评分和HJHS∶总体步态评分均降低；与基线相比，1年随访期按需治疗组重型HA患者最大Gilbert评分显著降低（*P*＝0.02）；按需治疗组HA患者HJHS∶总体步态评分显著降低（*P*＝0.008）；预防性治疗组HA患者平均Gilbert评分的降低幅度高于按需治疗组（28.6％对18.2％）。

**表4:** 不同疾病严重程度患者Gilbert评分和HJHS∶总体步态评分

描述	预防性治疗组				按需治疗组			
	基线（26例）	1年（13例）	统计量	*P*值	基线（36例）	1年（9例）	统计量	*P*值
最大Gilbert评分	5.9±3.0	4.8±3.4	1.00	0.33	6.5±3.9	5.3±1.5	1.46	0.15
重型	4.8±2.5	3.7±3.5	0.81	0.43	8.6±4.6	5.0±1.2	2.51	0.02
中间型	8.2±2.6	7.2±0.5	1.09	0.30	5.6±6.2	5.8±1.9	−0.17	0.87
平均Gilbert评分	5.0±2.5	3.6±2.8	1.56	0.13	5.5±3.2	4.5±1.5	1.36	0.18
重型	3.7±1.6	2.7±2.8	1.36	0.18	5.9±3.4	3.8±1.0	1.95	0.07
中间型	7.5±1.9	5.6±1.9	1.74	0.13	5.3±3.2	5.4±1.7	−0.09	0.93
HJHS∶总体步态评分	1.5±1.6	0.9±1.1	1.25	0.23	1.9±1.7	0.4±0.6	3.0	0.008
重型	1.0±1.3	1.2±1.5	−0.29	0.79	2.7±1.2	0.0±0.0	NA	NA
中间型	2.3±1.7	0.7±0.6	3.04	0.01	1.8±1.8	0.4±0.6	2.37	0.03

**注** HJHS：血友病关节健康评分；重型：凝血因子Ⅷ（FⅧ）活性<1％；中间型：FⅧ活性1％～5％

四、出血情况

与基线相比，预防性治疗组、按需治疗组HA患者1年随访期ABR评分的平均值（3.70对7.78、22.98对29.92）均降低，且1年随访期预防性治疗组患者AJBR评分的平均值（2.70对3.91）低于基线（表5）。与基线时相比，1年随访期预防性治疗组、按需治疗组ABR＝0的患者比例（49.3％对24.8％，18.8％对15.1％）均高于基线，且预防性治疗组1年随访期AJBR＝0的患者比例高于基线（56.7％对34.5％）（表5）。如表6所示，与基线相比，预防性治疗组HA患者1年随访期ABR评分显著降低（*P*＝0.010），尤其是重型HA患者（*P*＝0.006）。预防性治疗组儿童HA患者1年随访期ABR评分低于基线（*P*＝0.03，表7）。接受预防性治疗的低剂量重型HA患者1年随访期ABR评分低于基线（*P*＝0.04，表8）；接受预防性治疗的中剂量组HA患者1年随访期ABR评分显著低于基线（*P*＝0.015），尤其是重型HA患者（*P*＝0.048）。

**表5:** 接受Octocog alfa治疗血友病A患者基线和1年随访期出血情况

指标	基线		1年随访期	
	预防性治疗（113例）	按需治疗（53例）	预防性治疗（67例）	按需治疗（32例）
出血事件［例（％）］	85（75.2）	45（84.9）	36（53.7）	28（87.5）
出血原因［次数/总次数（％）］				
外伤	39/879（4.4）	18/1586（1.1）	44/261（16.9）	35/757（4.6）
自发出血	114/879（13.0）	58/1586（3.7）	44/261（16.9）	74/757（9.8）
原因不明	726/879（82.6）	1510/1586（95.2）	173/261（66.3）	648/757（85.6）
ABR评分				
中位数（*Q*_1_, *Q*_3_）	2.22（1.00, 10.00）	20.00（3.00, 47.00）	0.87（0.00, 5.21）	20.69（3.62, 36.60）
均数（95％ *CI*）^a^	7.78（5.06, 10.50）	29.92（20.58, 39.26）	3.70（1.86, 5.54）	22.98（14.61, 31.35）
ABR＝0［例数（％）］	28（24.8）	5（15.1）	33（49.3）	6（18.8）
AJBR评分				
中位数（*Q*_1_, *Q*_3_）	1.00（0.00, 4.00）	4.00（1.00, 15.00）	0.00（0.00, 3.13）	11.11（1.14, 28.52）
均数（95％ *CI*）^a^	3.91（2.13, 5.69）	10.85（6.19, 15.51）	2.70（1.31, 4.09）	15.84（10.64, 21.04）
AJBR＝0［例数（％）］	39（34.5）	12（22.6）	38（56.7）	7（21.9）

**注** N：具有非缺失数据的患者数；ABR：年化出血率；AJBR：年化关节出血率；^a^基于负二项分布的广义线性混合效应模型进行事后ABR和AJBR分析，并使用正态逼近法对95％ *CI*进行估计

**表6:** 不同疾病严重程度血友病A患者ABR和AJBR评分（*x*±*s*）

组别	预防性治疗组				按需治疗组			
	基线（113例）	1年（67例）	统计量	*P*值	基线（53例）	1年（32例）	统计量	*P*值
ABR评分	7.78±14.60	3.70±7.54	2.47	0.010	29.92±34.32	22.98±23.22	1.11	0.27
重型	8.98±18.16	2.14±3.36	2.84	0.006	27.53±29.81	24.41±19.57	0.34	0.74
中间型	6.59±9.15	5.63±10.40	0.42	0.680	27.97±32.07	22.34±25.10	0.73	0.47
AJBR评分	3.91±9.53	2.70±5.70	1.07	0.290	10.85±17.11	15.84±14.42	−1.44	0.15
重型	4.57±11.07	1.73±2.86	1.89	0.060	13.16±21.42	20.85±17.21	−1.05	0.31
中间型	3.27±7.56	3.90±7.81	−0.35	0.720	7.73±9.74	13.56±12.75	−1.82	0.08

**注** ABR：年化出血率；AJBR：年化关节出血率；重型：凝血因子Ⅷ（FⅧ）活性<1％；中间型：FⅧ活性1％～5％

**表7:** AHEAD研究中国组不同年龄血友病A患者ABR和AJBR评分（*x*±*s*）

组别	预防性治疗组				按需治疗组			
	基线（113例）	1年（67例）	统计量	*P*值	基线（53例）	1年（32例）	统计量	*P*值
ABR评分								
儿童	4.73±6.82	2.10±3.12	2.22	0.03	48.33±45.37	14.74±14.69	1.22	0.33
青少年	4.85±7.11	1.72±2.36	1.51	0.15	22.70±42.78	18.83±12.43	0.27	0.79
成人	11.88±20.37	5.30±9.88	1.95	0.06	30.35±31.70	25.43±26.63	0.65	0.52
AJBR评分								
儿童	1.65±2.52	1.20±2.50	0.67	0.51	6.33±7.57	9.12±9.46	−0.40	0.71
青少年	1.54±1.85	1.72±2.36	−0.22	0.83	11.40±22.01	18.83±12.43	−0.88	0.39
成人	7.00±13.84	4.04±7.38	1.26	0.21	11.05±16.55	16.99±16.15	−1.37	0.18

**注** ABR：年化出血率；AJBR：年化关节出血率；儿童：2岁～<12岁，青少年：12岁～<18岁，成人：≥18岁

**表8:** 不同剂量预防性治疗组血友病A患者年化出血率（ABR）评分（*x*±*s*）

组别	低剂量组				中剂量组			
	基线（58例）	1年（33例）	统计量	*P*值	基线（39例）	1年（26例）	统计量	*P*值
整体	7.09±10.59	4.17±9.15	1.38	0.17	8.26±14.82	2.03±3.47	2.52	0.015
重型	7.41±10.89	2.61±3.56	2.12	0.04	9.83±18.41	1.65±3.12	2.09	0.048
中间型	6.93±10.65	5.83±12.63	0.30	0.77	6.00±7.10	2.63±4.06	1.54	0.140

**注** 低剂量组：Octocog alfa每周剂量<45 IU/kg；中剂量组：Octocog alfa每周剂量45～75 IU/kg；高剂量（Octocog alfa每周剂量>75 IU/kg）组患者例数<3，未纳入分析

五、不良事件发生情况

试验期间25例（14.9％）患者发生了39次AE，较为常见的AE为上呼吸道感染（6例次）、流行性感冒（3例次）、牙痛（2例次）和发热（3例次）等。8例患者报告了8次SAE，分别为颅内出血（2例次）、肛瘘（1例次）、骨折（1例次）、阑尾炎（1例次）、抗FⅧ抗体阳性（1例次）、血友病性关节炎（1例次）和过敏性紫癜（1例次），仅1例患者报道的抗FⅧ抗体阳性与治疗相关。在试验的主要阶段，1例（0.6％）患者检测到FⅧ抑制物（≥0.6 BU/ml）。

## 讨论

HA患者的关节出血通常引起滑膜组织和关节软骨病变，导致血友病关节病。血友病关节病是HA患者关节退化和致残的主要原因[16]。Gilbert评分常用于评估HA患者的关节功能和关节健康状态，HJHS∶总体步态评分主要评估患者步态的整体流畅性、稳定性和对称性等[17]–[18]。一项前瞻性研究纳入20例接受重组FⅧ治疗的重型HA患者，显示预防性治疗组患者的Gilbert评分中位数低于按需治疗组（18对25）[19]。本研究中发现预防性治疗组和按需治疗组患者接受Octocog alfa治疗1年后Gilbert评分均降低。预防性治疗组患者平均Gilbert评分从基线的5.03降低至1年后的3.59，按需治疗组由5.5降低至4.5，预防性治疗组患者平均Gilbert评分的降低幅度高于按需治疗组（28.6％对18.2％）。与基线相比，预防性治疗组中间型HA患者1年随访期的HJHS∶总体步态评分显著降低（*P*＝0.01），按需治疗组患者HJHS∶总体步态评分显著降低（*P*＝0.008）。表明HA患者接受重组凝血因子Ⅷ预防性治疗后关节活动能力的改善高于按需治疗，但预防性治疗和按需治疗对HA患者的步态均有显著改善。

针对HA患者实施合理且有效的止血治疗策略是确保他们健康和生活质量的关键。预防性治疗的目的在于防止患者潜在的出血风险，按需治疗的目的在于应对患者的急性出血事件，二者都是为了帮助患者保持正常的生活节奏和减少因出血导致的身体损伤和心理压力[20]–[21]。HERO调查研究显示，与发达国家（565例）相比，我国HA患者（110例）就业率（45.6％对63.1％）、预防性治疗（4.1％对36.8％）和治疗依从性（6.2％对40.5％）比例均较低，且每年需要治疗的出血次数更多（平均29.4次对15.4次）[22]。ABR反映了HA患者1年内总出血次数，AJBR专注于关节出血频率，ABR和AJBR共同指导医生优化治疗策略，是衡量HA患者疾病控制水平和治疗效果的关键指标[23]–[24]。临床研究显示，与接受rFⅧ按需治疗相比，HA患者进行预防性治疗后中位ABR显著降低（1.9对41.5，*P*<0.0001）[25]。系统评价的结果显示，相比于其他标准半衰期rFⅧ产品（lonoctocog alfa）和延长半衰期rFⅧ产品（efmoroctocog alfa），Octocog alfa可达到与其相似的止血效果，能够成为预防HA患者出血的有效治疗选择[26]。本研究结果显示，与基线相比，接受Octocog alfa预防性治疗的HA患者1年随访期平均ABR显著降低（3.70对7.78，*P*＝0.010），尤其是重型HA患者（2.14对8.98，*P*＝0.006）和儿童HA患者（2.1对4.73，*P*＝0.03）。同时，1年随访期预防性治疗组患者AJBR的平均值（2.70对3.91）降低和AJBR＝0的患者比例升高。英国血友病中心医生组织指南推荐重型儿童HA患者使用FⅧ浓缩物进行预防性治疗，以防止关节血肿和其他出血事件，且指南推荐预防性治疗的输注剂量应为每48 h注射1次FⅧ浓缩物（25～50 IU/kg）[27]。本研究显示与基线相比，接受Octocog alfa预防性治疗的低剂量组（平均每周注射Octocog alfa<45 IU/kg）重型HA患者1年随访期ABR评分显著降低（*P*＝0.04）；中剂量组（平均每周注射Octocog alfa为45～75 IU/kg）HA患者1年随访期ABR评分显著降低（*P*＝0.015）。以上结果显示接受Octocog alfa预防性治疗的患者在1年随访期出血情况显著改善。Octocog alfa预防性治疗的注射剂量低于指南推荐的标准，但仍能有效控制出血。

rFⅧ的安全性在临床上存在争议，重型HA患者接受rFⅧ治疗后抑制物的发生率约为30％，中间型或轻型HA患者的发生率为3％～13％[28]–[29]。抑制物发生率可能与rFⅧ产品类型、患者年龄和治疗策略等多种因素有关[30]。本研究168例患者报告的AE发生率为14.9％，4.8％的患者报告了严重AE，其中仅1例（0.6％）患者报告的抗FⅧ抗体阳性与治疗相关，表明患者接受Octocog alfa治疗后的抑制物发生率较低，安全性较好。

综上所述，本研究发现，无论是接受Octocog alfa预防性治疗还是按需治疗，HA患者的关节活动能力和出血情况均有改善。接受预防性治疗的患者ABR的改善具有统计学显著性，尤其是重型HA患者和儿童HA患者。Octocog alfa治疗期间患者的抑制物发生率较低，证明其具有良好的安全性。

本研究为非干预性研究，存在明显的局限性：①由于未对受试者实施人为干预，难以确立Octocog alfa治疗与HA患者临床结局间的直接因果关系。②1年随访期内出现数据的缺失，虽然分析基于可用数据，但仍可能引入偏倚，影响结果的精确性和全面性。③本研究未设置对照组，也缺乏Octocog alfa的药代动力学数据。期待未来有新的临床研究填补这方面内容的空白，以期更好地指导临床实践。
